# Frequency of Tumor Necrosis Factor-α, Interleukin-6, and Interleukin-10 Gene Polymorphisms in Mexican Patients with Diabetic Retinopathy and Diabetic Kidney Disease

**DOI:** 10.3390/pathophysiology32020014

**Published:** 2025-04-09

**Authors:** Patricia Elvira Sánchez-Valencia, Juan Daniel Díaz-García, Margarita Leyva-Leyva, Fabiola Sánchez-Aguillón, Nelly Raquel González-Arenas, Jesús Guillermo Mendoza-García, Erika Karina Tenorio-Aguirre, Mercedes Piedad de León-Bautista, Aurora Ibarra-Arce, Pablo Maravilla, Angélica Olivo-Díaz

**Affiliations:** 1 División de Medicina Interna, Hospital General “Dr. Manuel Gea González”, Calzada de Tlalpan 4800, Col. Sección XVI, Mexico City 14080, Mexico; patysanchezval@gmail.com (P.E.S.-V.); judandigar@gmail.com (J.D.D.-G.); bazluhrmann03@hotmail.com (J.G.M.-G.); dra_erikari@yahoo.com.mx (E.K.T.-A.); 2 Departamento de Biología Molecular e Histocompatibilidad, Hospital General “Dr. Manuel Gea González”, Calzada de Tlalpan 4800, Col. Sección XVI, Mexico City 14080, Mexico; margaritaleyvaleyva@yahoo.com.mx (M.L.-L.); ofalabi_j@hotmail.com (F.S.-A.); auroraibarraarce@yahoo.com (A.I.-A.); 3 Departamento de Ecología de Agentes Patógenos, Hospital General “Dr. Manuel Gea González”, Calzada de Tlalpan 4800, Col. Sección XVI, Mexico City 14080, Mexico; nelly_raquel@hotmail.com (N.R.G.-A.); maravillap@yahoo.com (P.M.); 4 Escuela de Medicina, Universidad Vasco de Quiroga, Morelia 58090, Mexico; dramercedespiedad@gmail.com; 5 Laboratorio de Enfermedades Infecciosas y Genómica (INEX LAB), Morelia 58280, Mexico

**Keywords:** type 2 diabetes, polymorphisms, tumor necrosis factor-α, interleukin-10, interleukin-6, association

## Abstract

Background/Objectives: Two of the microvascular complications in type 2 diabetes (T2D) are diabetic retinopathy (DR), which is the most common cause of non-traumatic blindness, and diabetic kidney disease (DKD); the latter generally requires renal replacement therapy. The aim of the present study was to determine the frequency of polymorphisms of Tumor Necrosis Factor-α, interleukin-6, and interleukin-10 (*TNF-α*, *IL-10,* and *IL-6*), as well as to describe the clinical and laboratory characteristics of T2D association with these microvascular complications. Methods: This study included 203 patients with T2D, of which 102 had microvascular complications: 95 with DR, 50 with DKD, and 15 with diabetic neuropathy (the latter were not included in the statistical analysis); those with T2D without confirmed microvascular complications were considered as controls. Clinical and laboratory data were collected from the patient’s medical records. Polymorphism typing of *TNF-α* rs361525 and rs1800629 and *IL-10* rs1800872 and rs1800871 were obtained using MALDI-TOF MS. *IL-10* rs1800896 and *IL-6* rs1800795 were typed using a quantitative real-time polymerase chain reaction. Results: The results of age, HbA1c, fasting glucose, and arterial hypertension are significantly associated in every group. The *TNF-α* rs1800629A allele and *TNF-α* rs1800629G/A genotype were associated with microvascular complications and DR. For *IL-10*-rs1800896, all the models were associated in DKD. The *TNF-α* rs361525-rs1800629GA haplotype was associated with microvascular complications and DR, while the *IL-10* haplotype, rs1800872-rs1800871-rs1800896 GGC, showed susceptibility in every group. Conclusions: Our results show the contributions of the variants of these cytokines to these microvascular complications, but more studies are required to reach relevant conclusions.

## 1. Introduction

Type 2 diabetes (T2D) is an increasingly common metabolic disease presenting different microvascular complications [1]. The consequence of insulin resistance is hyperglycemia, and the chronicity of this affects various organs such as the eyes, nerves, kidneys, and heart [2]. Chronic hyperglycemia manifests as microvascular complications, and even when controlled, the morbidity associated with these complications continues to increase. Impairment of microvascular function can arise even before hyperglycemia and vascular pathological changes manifest. Diabetes induces characteristic changes in the microvasculature, affecting the basement membrane of capillaries, including arterioles in the glomeruli, retina, myocardium, skin, and muscle [2]. In diabetes patients, clinical investigations have shown a link between the accumulation of advanced glycation end products (AGEs) and vascular damage since AGEs play a central role in the pathophysiology of diabetes complications, including retino- and nephropathies, even identifying the presence of autoantibodies against glycated fibrinogen [3].

Diabetic retinopathy (DR) is a frequent microvascular complication of T2D and the most common cause of non-traumatic blindness, occurring in approximately 18.5% to 34.6% of people with diabetes, especially among patients aged 60 to 69 years. This risk increases considerably with the duration of the disease [4,5].

Multiple studies have reported that DR is a multifactorial disease whose development and progression result from the interaction between genetic and clinical risk factors. Although there are multiple metabolic alterations involved, the exact mechanism is not known. Clinical risk factors for diabetic retinopathy include the duration of diabetes, glycemic control, concomitant hypertension, and other environmental factors. However, some patients who do not control blood glucose and blood pressure do not develop DR and, in contrast, patients with good glucose control and without hypertension can develop DR [6].

Diabetic kidney disease (DKD) is another microvascular complication caused by T2D and is a common chronic kidney disease that requires renal replacement therapy. Classically, it has been defined by the presence of proteinuria at 0.5 g/24 h. This stage has been called overt nephropathy, clinical nephropathy, proteinuria, or macroalbuminuria [7]. Some estimates of the rate of development of nephropathy in type 2 diabetes indicate that microalbuminuria is relatively common, occurring in almost one-quarter of patients 10 years after the diagnosis of diabetes, whereas macroalbuminuria and renal replacement therapy are less common [8]. The pathogenesis of DKD development and progression is complex and multifactorial, involving many pathways and mediators [9].

Cytokines are important mediators in the immune system and its response, and due to an inadequate balance, they can largely regulate the predisposition to diseases [10]. As an approach to understanding and controlling T2D and its microvascular complications, several serological biomarkers related to inflammation have been studied, such as tumor necrosis factor-α (TNF-α), which is the cytokine most related to DR [11] and also correlates with inflammation in various conditions, for example with the vascularization of radicular cysts [12]. On the other hand, interleukin (IL)-6, whose levels are elevated in the vitreous humor of patients with DR, may be a predictor of proliferative DR [13], while IL-10, a regulatory cytokine, has been associated as a negative risk factor for DR [14].

Some studies suggest an association between single nucleotide polymorphisms (SNPs) of these cytokines and the microvascular complications that occur in T2D. An association between proliferative DR and *TNF-α* -308G/A (rs1800629) polymorphism has been described [15], whereas non-proliferative DR is associated with *IL-10* -1082G/A (rs1800896) [16]. The *IL-10* -592C/A (rs1800872) [17], *IL-10* -819T/C (rs1800871) [18], and *IL-6* -174G/C (rs1800795) [19] polymorphisms have been found to be associated with DKD in several populations.

There is still controversy surrounding the role that cytokine gene polymorphisms play in diabetes complications. Some are associated with susceptibility, while others may be considered protective factors. Therefore, the aim of the present study was to determine the frequency of polymorphisms of *TNF-α*, *IL-10,* and *IL-6*, as well as to describe the clinical characteristics of Mexican patients with T2D who developed DR and DKD compared to T2D without these microvascular complications.

## 2. Materials and Methods

### 2.1. Samples and Data Collection

An observational and cross-sectional case-control study was conducted from March to August 2020. Two hundred and three Mexican patients with T2D, 128 men and 75 women, from 24 to 88 years with a mean of 56.13 ± 13.73 (Standard deviation; SD) years were included, of which 102 had microvascular complications: 95 had DR, 50 had DKD, and 15 had diabetic neuropathy (the latter were not included in the statistical analysis); those with T2D without confirmed microvascular complications were considered controls. All participants were patients of the Internal Medicine Service of the Hospital General “Dr. Manuel Gea González”.

T2D diagnosis was based on the American Diabetes Association (ADA) [20]. DR was diagnosed by expert ophthalmologists, and DKD was defined as the presence of microalbuminuria, determined by urinary albumin of 30–300 mg/g creatinine in laboratory analysis, supervised by the internist [21]. The results of arterial hypertension (AHT), LDL cholesterol, HDL cholesterol, and triglycerides were taken from the most recent patients’ medical records according to ADA criteria. Clinical and laboratory data, including age, weight, height, body mass index (BMI), onset of T2D, fasting glucose, glycosylated hemoglobin (HbA1c), and smoking history, were also obtained from the medical history.

This study complies with current health laws in Mexico and the Helsinki Declaration of 1964 and its subsequent modifications. The Research Ethics and Research Committees of the General Hospital “Dr. Manuel Gea González” approved this study on June 29, 2020, with the reference number 12-21-2020. Written informed consent was obtained from each participant prior to sampling.

### 2.2. Genotyping

DNA was obtained from 10 mL of EDTA-peripheral blood using proteinase K and phenol/chloroform extraction [22]. DNA was stored at −20 °C until use.

Six polymorphisms corresponding to *TNF-α* (rs361525 and rs1800629), *IL-10* (rs1800872, rs1800871, and rs1800896), and *IL-6* (rs1800795) were typed, which were selected for their association with microvascular complications in T2D.

The 4 PCR primer pairs and 4 probes for *TNF-α* rs361525, *TNF-α* rs1800629, *IL-10* rs1800872, and *IL-10* rs1800871 were designed using MassARRAY Assay Design v 3.1 software (Sequenom, Inc., San Diego, CA, USA) with the following design parameters: locus-specific primer sizes between 12 and 30 nucleotides, average sizes of amplicons between 80 and 120 base pairs (bp), an annealing temperature between 45 and 100 °C, optimal robustness of 0.8 in loop formation, dimers and false amplification primers, average robustness in dimer formation and false primer extension of 0.5, a spectrometric range of 4 to 10 kDa, a separation distance between SNPs of 15 Da, and a maximum multiplexing number of 8 SNPs.

The method of MALDI-TOF mass spectrometry (MALDI-TOF MS) of DNA molecules in the format of an iPLEX Assay implemented on the Sequenom MassARRAY4 platform (Agena Bioscience, San Diego, CA, USA) was performed as described by Trifonova et al. [23]. Genotyping of the SNPs rs1800629, rs361525, rs1800872, and rs1800871 was performed in a 384-well plate format on the MassARRAY System Analyzer 4.0.163 (Sequenom, San Diego, CA, USA), which combines polymerase chain reaction (PCR) and matrix-assisted laser desorption/ionization time-of-flight mass spectrometry technologies (MALDI-TOF MS). The PCR products were subjected to uric acidification and a primer single-base extension reaction. Alleles were then detected by Matrix-Assisted Laser Desorption/Ionization Time-Of-Flight (MALDI-TOF) mass spectrometry, and mass spectra analysis was conducted with Mass Array Typer 4.0 software (Agena Bioscience, San Diego, CA, USA).

Genotyping for *IL-10* rs1800896 and *IL-6* rs1800795 gene polymorphisms was conducted using the quantitative real-time polymerase chain reaction (RT-qPCR) with the TaqMan probes described in Table 1. A volume of 15 μL of the qPCR reaction mixture was prepared, consisting of 0.5 μM of TaqMan probes, 7.5 μL of LightCycler 480 Probes Master (Roche Diagnostics), 2.5 μL of RNasefree water, and 2 μL of DNA (50 ng/μL). The qPCR procedure was carried out on a LightCycler480 system (Roche, Germany) with the following conditions: 95 °C for 10 min, followed by 45 cycles of 95 °C for 15 s and 60 °C for 1 min. Data analysis was conducted using the LightCycler 480 software. The primers and probes are listed in Table 1.

### 2.3. Statistical Analyses

The categorical clinical variables of patients and control subjects were compared using the X^2^ test or Fisher’s test when appropriate, and continuous clinical variables were compared using Student’s *t*-test or the Mann–Whitney U-test when appropriate. Continuous (dummy variables) and categorical variables were analyzed using SPSS v20.0 software (SPSS Inc., Chicago, IL, USA). Allele (AFs) and genotype (GFs) frequencies were calculated by direct counting. Differences in frequencies between groups were compared using a Pearson Chi-squared test, considering *p* ≤ 0.05 as the minimum level of significance. A two-tailed Fisher’s exact test was used when the expected frequency in at least one cell was 5 or less. Relative risk was calculated as an odds ratio (OR). Ninety-five percent confidence intervals (95% CI) were obtained using Cornfield’s approximation. Haplotypes, Hardy–Weinberg equilibriums, and linkage disequilibrium were determined by the confidence interval method using Haploview 4.2 software [24]. To establish the most informative model of Mendelian association (dominant, co-dominant, recessive, over-dominant, or log additive), the online SNPStats program was used. An unconditional logistic regression was performed using Wald’s backward steps.

## 3. Results

### 3.1. Clinical Features

Comparisons among the different groups are shown in Table 2. Age, HbA1c, fasting glucose, and AHT are significantly associated in every group. The male gender was associated with susceptibility to DR and LDL with the two microvascular complications, whilst BMI and weight were associated with microvascular complications and DR. Variables such as diet, lifestyle, or genetic ancestry were not included because that information was not available. However, patient medication use was as follows: metformin 88.18%; glibenclamide 70.44%; DPP4 inhibitors 26.11%; GLT2 inhibitors 5.42%; ACE inhibitors 28.08%; ARBs 16.26%; calcium channel blockers 14.29%; statins 50.25%; and fibrates 50.25%. These data were not included in the analysis.

### 3.2. Polymorphisms

The analysis of the polymorphisms (Table 3) showed an association of the TNF-α rs1800629A allele and the TNF-α rs1800629 G/A genotype with microvascular complications and DR. The IL-10-rs1800896C allele and the IL-10-rs1800896C/C genotype were associated in the codominant and recessive models with microvascular complications and DR. All the models were associated in DNP, but only codominant and recessive are shown.

Regarding rs1800795, only the association of the rs1800795G allele with protection against DNP was found, although the confidence interval reached the unit.

The haplotype TNF-α rs361525-rs1800629GA was associated with susceptibility in T2D with microvascular complications and DR, while TNF-α rs361525-rs1800629 GG was associated with protection in both groups. In the IL-10 haplotypes, rs1800872-rs1800871-rs1800896 GGC showed susceptibility in every group and IL-10 rs1800872-rs1800871-rs1800896 GGT showed protection against DR (Table 4).

Logistic regression unconditional by Wald backward steps showed an association between AHT, age, weight, HbA1c, rs1800896C/C, and rs1800896T/C and microvascular complications (Table 5). In DR, AHT, weight, rs1800896C/C, and rs1800896T/C were associated with susceptibility, while HbA1c was associated with DR susceptibility. For DKD, T2D time, BMI, and rs361525AG were related to protection.

## 4. Discussion

Since numerous associations between SNPs at different loci in the development of DR and DKD have been documented, these types of experimental approaches have helped to understand the pathophysiology of these diseases, e.g., the *GAS5* SNP rs145204276 Del/Del variant was more prevalent and correlated with a shorter disease duration of DM in DR patients from Taiwan [25]. The present study investigated certain polymorphisms of the cytokine genes *TNF-α, IL-10*, and *IL-6* in patients with T2D and their main microvascular complications as DR and DKD. As expected, statistically significant differences were found in older people with microvascular complications, both in the comparison of microvascular complications in general and in the classification of DR and DKD. While we know that there are ethnic differences among populations, we believe it is important to mention both the similarities and discrepancies with other studies. Our findings regarding age are concordant with other studies of DR [26,27,28,29,30], with the exception of Paine et al. [16] and Cilensek et al. [31], in which the average age of those without is older than the group with DR. It is also evident that the patients with DKD were older [18,19,28,32,33,34,35] (Supplementary Table S1).

In our data, in general, there were more men with microvascular complications than women, with this being statistically significant only in DR (Table 2). In some studies, there were more women than men [3,27,29,32], while in others, it was the other way around [16,26,30]. Only the study by da Silva Pereira et al. [29] showed an association between DR and women. In the study by Fathy et al. [35], there were more women than men and more men than women with and without DKD, respectively, showing statistical significance.

In our study, BMI was associated with DR, in agreement with the results of Rodrigues et al. [28] and Liu et al. [30], but there was no association with DKD. However, Ezzidi et al. [18] and Mtiraoui et al. [32] did find an association with DKD in their populations. In addition, Sikka et al.’s [36] results show an association with both.

Regarding LDL and HDL concentrations, in our study, we only found significant differences in LDL between patients with and without microvascular complications. Da Silva Pereira et al. [29] found an association between HDL and DR, unlike Sikka et al. [36], who did not find it. Moreover, Zhong et al. [37], Yoshioka et al. [26], Cilenšek et al. [31], and Liu et al. [30] analyzed the concentrations of both LDL and HDL and neither had significant differences in DR. On the other hand, Sikka et al. [36] found an association between DKD and HDL; however, in the studies by Arababadi et al. [38], Ezzidi et al. [18], Mtiraoui et al. [32], and Fathy et al. [35] there was no significance with LDL or HDL.

In our study, no significant differences were found between any of the groups in terms of triglyceride concentration, which coincides with the studies of Yoshioka et al. [26], da Silva Pereira et al. [29], Cilenšek et al. [31], Fathy et al. [35], and Zhong et al. [37]; only Mtiraoui et al. [32] found statistical significance with DKD.

All the HbA1c comparisons made in this study were significant, as in the works of Zhong et al. [37] and Yoshioka et al. [26] for DR and Ezzidi et al. [18], Rodrigues et al. [28], and Mtiraoui et al. [32] for DKD.

Regarding the presence of hypertension, in all the comparisons that were made in our study, there were significant differences, while da Silva Pereira et al. [29] and Liu et al. [30] only found significance with DR.

The results of the polymorphisms showed the association of the rs1800629A allele and the rs1800629G/A genotype with microvascular complications and DR, indicating that DR is the one with the greatest weight in the association. Only one study was found in which there was an association between the genotypes rs1800629G/A and rs1800629A/A and DKD [34], while in other studies, there was no association with any comorbidity [16,26,27,28,35,36,39]. Gao et al., [1] found a marginal association between rs1800629 and DR in a meta-analysis (the ORs [95% CI] of [GA vs. GG], [GA + AA] vs. GG, and [A vs. G] are 1.21 [1.04, 1.41], 1.20 [1.03, 1.39], and 1.14 [1.01, 1.30], respectively). The subgroup analysis indicated an enhanced association within the European population (for GA vs. GG: OR 1.27, 95% CI 1.06–1.51; for [GA + AA] vs. GG: OR 1.25, 95% CI 1.05–1.49; for A vs. G: OR 1.17, 95% CI 1.01–1.36 (Data not included in Supplementary Table S2)). Moemen et al. [40] mentioned that the G/G genotype of the -308 G/A polymorphism occurred more frequently in proliferative DR patients than in non-proliferative DR patients. Tiongco et al. [41] in a meta-analysis found that T2D individuals with the -308 G/A polymorphism in the TNF-α gene are more likely to develop DKD since they say that: “Significant associations were observed in the Asia subgroup for the co-dominant (OR: 2.03; 95% CI: 1.41–2.92; *p* = 0.0001) and recessive (OR: 1.73; 95% CI: 1.21–2.47; *p* = 0.002) models with homogeneity” (Data not included in Supplementary Table S2).

Although our results for the rs361525 SNP are apparently associated with the presence of microvascular complications and DR, not having patients with the rs361525G/A genotype means we were not able to calculate the confidence interval, so the result is not conclusive. The study by Paine et al. [16] did find an association of the rs361525A allele and the rs361525G/A and rs361525A/A genotypes with DR, as did the study by Hameed et al. [34] with the dominant genotype rs361525G/A+ rs361525A/A and DKD. Gao et al. [1] described an association between the whole population and DR (the OR [95% CI] of [GA vs. GG] is 1.55 [1.14, 2.11]). In the study by Fathy et al. [35], no association with DKD was found.

In the *IL-10* rs1800872 and rs1800871 polymorphisms, we did not find an association, which coincided with other studies [28,35,38], with the exception of one study that presented an association between rs1800871 and DKD [32].

However, in the IL-10 rs1800896 polymorphism, the rs1800896C allele was associated with all microvascular complications, whereas genotype analysis showed an association of rs1800896C/C with microvascular complications and DR in the codominant model (Table 3). In the literature, an association between rs1800896C/C genotype DR [16,31] and DR and DKD [28,35] was observed, while the rs1800896C allele was associated with DR [16,30,31] and DKD [35]. In some studies, there was no association with DKD [33].

Regarding the SNP rs1800795, we only found an association with the rs1800795C allele and DKD; the other apparent associations we found were not taken into account because the confidence interval could not be calculated. There was only one study in which the authors reported an association between the rs1800795G/C genotype and DR [42]; in the other studies, there was no association [16,28,34,35].

No reports of the TNF-α haplotypes studied here were found in the literature. Of the IL-10 haplotypes similar to those we reported, in the study by Fathy et al. [35], the CGG=GCC haplotype was associated with protection for DKD; the other two studies that analyzed haplotypes established no differences between the groups [28,32].

Although the study design only allows for establishing associations between genetic polymorphisms and complications, hypotheses can be raised about how they might influence the development of complications, considering what has been described in the literature.

TNF-α is a pro-inflammatory cytokine whose production is influenced by the polymorphisms present in the promoter region. In particular, the -238G allele is associated with high expression, while the -238A allele decreases it. Regarding position -308, while some describe the -308A allele as increasing transcription and TNF-α production, others suggest that this allele has no effect on TNF-α [43]. It has been demonstrated that TNF-α increases early during the course of DR, particularly in proliferative DR, where it is found in the extracellular matrix, endothelium, and vessel walls of the fibrovascular tissue of the eyes and is elevated in the vitreous. Treatment with high doses of anti-inflammatory agents reduces the amount of TNF-α in the diabetic retina [44]. We found significant elevation in the rs1800629A allele and the rs1800629GA (-308) genotype in patients with DR, which would suggest a possible increase in the intraocular production of TNF-α [45].

On the other hand, IL-10 is a regulatory cytokine since it inhibits the generation of proinflammatory cytokines, antigen presentation, and cell proliferation, whose polymorphisms influence its production, particularly rs1800896. [46]. In a Chinese population, the GG genotype and the G allele of the *IL-10* -1082G/A polymorphism, as in a Brazilian population, were associated with a lower risk of proliferative DR and DR, respectively [28,29,30]. However, in another study with the Chinese population, the TC genotype was found to be associated with the risk of proliferative DR [47] (Supplementary Table S2). In contrast, our results show an association of the C allele and the CC genotype with susceptibility to DR, which shows the existence of variation between different populations and the possible influence of other factors in the development of the disease. However, the fact that, in our work, the C allele and the CC genotype were associated with susceptibility to DR suggests that IL-10 production in the eye could be destabilized, allowing a local increase in proinflammatory cytokines, which could lead to ocular damage found in these patients.

IL-6 is a cytokine involved in immunomodulation, inflammation, increased vascular permeability, and hematopoiesis and stimulates cell proliferation, among other functions. A large number of studies have shown that IL-6 plays a key role in the development of retinal diseases, including DR. In the retina, several cell types, such as neurons, astrocytes, microglia, and endothelial cells, may produce IL-6 [48]. DR presents oxidative damage, inflammation, and pathological retinal vascular proliferation [49,50,51]. IL-6 production has been shown to be increased in patients with proliferative DR, and this may be related to the pathogenesis, severity, and prognosis of DR [52], as well as IL-6 levels in the aqueous humor [50]. Intraocular levels of IL-6 were found to be higher in the DR group than in the control group, but lower than in proliferative DR, with the *IL-6*-174 GC genotype marginally associated with proliferative DR [53]. Other studies describe the CC genotype with a higher frequency in DR [54] and the IL-6-174C allele associated with higher serum concentrations of IL-6 [55,56]. However, our results show no association with DR, likely due to the size of the sample analyzed and differences in the population’s genetic background.

With respect to DKD, the -308G/A rs1800629 polymorphism has been found to be associated with the onset of obesity [57] and insulin resistance in diabetes mellitus [58], suggesting that this polymorphism could be influencing the pathogenesis of DKD, as has been found in Asian populations, in whom the *TNF-α* -308A variant is associated with decreased nephropathy, but not in Caucasians [59]. In contrast, it has been seen that the level of TNF-α protein is much higher in patients with DKD than in healthy controls (*p* < 0.05). The TNF-α -308 G/A genotype and the A allele increase the risk of DKD (OR = 2.15, 95% CI = 1.08–4.30 and OR = 1.89, 95% CI = 1.10–3.26, respectively) in the Chinese population [60].

In the study by Umapathy et al., comparing diabetic patients with normoalbuminuria versus albuminuric patients (micro + macro), it was found that the *TNF-α* -308A allele was associated with a significant risk of albuminuria in the unadjusted OR. Similarly, the *TNF-α*-308AA genotype showed an association with albuminuria in the unadjusted OR. The plasma levels of TNF-α in albuminuric patients were significantly higher than in normoalbuminuric patients, indicating that the AA genotype is related to a higher production of this cytokine [61], as seen in other studies [62,63,64]. However, three other studies found no association between *TNF-α* -308 and DKD [65,66,67]. In our study, it was not possible to measure serum or plasma levels of TNF-α in patients; however, we did not find an association with DKD either.

In a meta-analysis by Peng et al., the *IL10* -1082A/G polymorphism was found to be significantly associated with an increased risk of DKD in both large and small sample studies, and it was concluded that this polymorphism might contribute to DKD susceptibility [68]. Similarly, Yin et al. found that the *IL-10* -1082 genotype AA was associated with an elevated risk of DKD compared with the GG genotype in all models [69]. Our results differ from the previous study since the association with DKD was observed in the CC (GG) genotype in all models. In patients from western Mexico, no association was found with the alleles or genotype of SNPs -1082 A/G (rs1800896), -819T/C (rs1800871), and -592A/C (rs1800872), but there was an association between the ATC (*p* = 0.0007) and GTA (*p* = 0.022) haplotypes (-1082A/-819T/-592C) and DKD. Our study also presents a haplotype associated with DKD, which is CGG, presented in the same order as in the article by Chavarria-Buenrostro et al. [70] (Supplementary Table S2).

In a meta-analysis of the *IL-6* -174G/C polymorphism and the risk of DKD, which evaluated whether this polymorphism may affect early DKD, Cui et al. found an association between the allelic and recessive genetic models (G vs. C: OR = 1.10, 95% CI 1.03–1.18, *p* = 0.006; GG vs. CC+GC: OR = 1.11, 95% CI 1.02–1.21, *p* = 0.016) [71]. However, some studies have shown no association between *IL-6* -174G/C polymorphism and DKD risk [28,34,54,72,73,74], while others show significant associations [19,75,76,77]. Our results showed a trend of the G allele protecting against DKD; however, we know that the sample size is too small to reach more precise conclusions.

## 5. Conclusions

In this study, the clinical data of T2D patients who presented microvascular complications, such as DR and DKD, as well as TNFα, IL-10, and IL-6 polymorphisms, were compared. One of the main limitations of the study is the sample size, which reduces the power of the statistical test, so a larger sample size is necessary to obtain more robust results. Another limitation was that due to the COVID-19 pandemic, it was not possible to collect a larger number of samples. However, despite these limitations, our results show that variants of these cytokines may be related to microvascular complications. Further studies are required to determine the involvement of these SNPs in DR and DKD.

## Figures and Tables

**Table 1:** Primers and probes for SNP typing of *TNF-α*, *IL-10,* and *IL-6*.

SNP ID	Forward and Reverse Primer ID	Forward and Reverse Primer Sequence	Extended Primer ID	Extended Primer Sequence
*TNF-α* rs361525_W1	rs361525_W1_F	ACGTTGGATGCACACAAATCAGTCAGTGGC	rs361525_W1_E	cccctACCCCCCTCGGAATC
	rs361525_W1_R	ACGTTGGATGTCTCGGTTTCTTCTCCATCG		
*TNF-α* rs1800629_W1	rs1800629_W1_F	ACGTTGGATGGCATCCTGTCTGGAAGTTAG	rs1800629_W1_E	AGGCTGAACCCCGTCC
	rs1800629_W1_R	ACGTTGGATGCTGATTTGTGTGTAGGACCC		
*IL-10* rs1800872_W1	rs1800872_W1_F	ACGTTGGATGTCCTCAAAGTTCCCAAGCAG	rs1800872_W1_E	cccccGACTGGCTTCCTACAG
	rs1800872_W1_R	ACGTTGGATGAAGAGGTGGAAACATGTGCC		
*IL-10* rs1800871_W1	rs1800871_W1_F	ACGTTGGATGATGCTAGTCAGGTAGTGCTC	rs1800872_W1_E	cccccGACTGGCTTCCTACAG
	rs1800871_W1_R	ACGTTGGATGGTACAGTAGGGTGAGGAAAC		
SNP ID	Forward and reverse Primer ID	Forward and Reverse Primer Sequence	Labelled probes	
*IL-10* rs1800896	rs1800896-F	ATGGAGGCTGGATAGGAGGT	rs1800896-T	[HEX]-CCTACTTCCCCTTCCCAAAG-[MGB]
	rs1800896-R	CACACACACACACAAATCCAAG	rs1800896-C	[FAM]-CCTACTTCCCCCTCCCAAAG-[MGB]
*IL-6* rs1800795	rs1800795-F	CGACCTAAGCTGCACTTTTCC	rs1800795-Wild-type	[Cy5]TTGTGTCTTGCGATGCTA[BHQ-2]
	rs1800795-R	AGATTGTGCAATGTGACGTCCTT	rs1800795-Mutant	[FAM]TTGTGTCTTGCCATGCTA[BHQ-1]

**Table 2:** Statistical differences in clinical and laboratory characteristics between diabetic patients and their microvascular complications.

Variable	T2D with Microvascular Complications n = 102	T2D Without Microvascular Complications n = 101	Statistical Significance &	T2D with DR n = 95	T2D Without DR n = 108	Statistical Significance &	T2D with DKD n = 50	T2D Without DKD n = 153	Statistical Significance &
Age, years	***58.53 ± 12.53***	***53.71 ± 14.50***	***0.012***	***58.18 ± 12.25***	***54.33 ± 14.73***	***0.046***	***61.36 ± 8.47***	***54.42 ± 14.68***	***6.45 × 10^−5^***
Sex									
Female (%)	33 (32.4)	43 (42.6)	0.132	***28 (29.5)***	***47 (43.5)***	***0.042***	13 (26.0)	62 (40.5)	0.065
Male (%)	69 (67.6)	58 (57.4)		***67 (70.5)***	***61 (56.5)***		37 (74.0)	91 (59.5)	
BMI (Kg/m^2^)	***28.46 ± 4.22***	***25.03 ± 3.22***	***6.60 × 10^−10^***	***28.40 ± 4.22***	***25.30 ± 3.46***	***5.15 × 10^−8^***	26.62 ± 3.63	26.80 ± 4.28	0.788
Weight (Kg)	***79.56 ± 15.76***	***67.23 ± 6.61***	***2.43 × 10^−11^***	***80.10 ± 16.03***	***67.55 ± 6.88***	***9.52 × 10^−11^***	73.70 ± 11.00	73.33 ± 14.34	0.850
T2D time (years)	12.87 ± 5.28	14.27 ± 5.15	0.058	13.29 ± 5.18	13.81 ± 5.32	0.490	13.66 ± 4.68	13.54 ± 5.43	0.885
DR time (years)				5.57 ± 2.74					
DKD time (years)							3.06 ± 1.65		
HbA1c (%)	***9.09 ± 2.06***	***8.00 ± 2.35***	***5.47 × 10^−4^***	***9.19 ± 2.06***	***7.98 ± 2.30***	***1.19 × 10^−4^***	***9.69 ± 2.48***	***8.17 ± 2.07***	***2.82 × 10^−5^***
Fasting glucose (mg/dL)	***152.43 ± 33.99 (8.46 mmol/L)***	***135.69 ± 23.35 (7.53 mmol/L)***	***6.47 × 10^−5^***	***153.85 ± 33.95 (8.54 mmol/L)***	***135.53 ± 23.71 (7.52 mmol/L)***	***1.93 × 10^−5^***	***155.64 ± 34.84 (8.64 mmol/L)***	***140.33 ± 27.76 (7.79 mmol/L)***	***6.10 × 10^−3^***
LDL (mg/dL)	***89.32 ± 28.22***	***97.74 ± 28.51***	***0.036***	89.44 ± 28.53	97.08 ± 28.34	0.057	91.68 ± 26.40	94.11 ± 29.36	0.604
HDL (mg/dL)	42.84 ± 11.41	43.25 ± 9.15	0.777	43.11 ± 11.59	42.98 ± 9.13	0.9322	41.84 ± 10.92	43.43 ± 10.13	0.345
Triglicerids (mg/dL)	204.38 ± 137.70	190.27 ± 84.51	0.379	206.37 ± 141.79	189.44 ± 82.88	0.3090	199.24 ± 137.35	196.75 ± 106.21	0.907
AHT									
Yes (%)	***62 (60.8)***	***30 (29.7%)***	***8.67 × 10^−6^***	***58 (61.1)***	***34 (31.5%)***	***2.41 × 10^−5^***	30 (60.0)	***62 (40.5%)***	***0.016***
No (%)	***40 (39.2)***	***71 (70.3%)***		***37 (38.9)***	***74 (68.5%)***		20 (40.0)	***91 (59.5%)***	
Smoking									
Yes (%)	42 (41.2)	35 (34.7)	0.338	40 (42.1)	37 (34.3)	0.295	23 (46.0)	54 (35.3)	0.176
No (%)	60 (58.8)	66 (65.3)		55 (57.9)	71 (65.7)		27 (54.0)	99 (64.7)	

& Comparisons between patients and control subjects were made using Student’s *t*-test for continuous variables and X^2^-test for categorical variables. Characters in bold and italics indicate association. T2D: Type 2 diabetes mellitus. DR: Diabetic retinopathy. DKD: Diabetic nephropathy. BMI: Body mass index. HbA1c: Glycosylated hemoglobin. AHT: Arterial hypertension. LDL: Low-density lipids. HDL: High-density lipids.

**Table 3:** Genotype and allele frequency of *TNF-α*, *IL-10,* and *IL-6* polymorphisms in T2D patients with microvascular complications (cases) and without microvascular complications (controls).

		T2D with VS. Without Microvascular Complications				T2D with VS. Without DR				T2D with VS. Without DKD			
Polymorphisms	Allele/Genotype	Cases (%)	Controls (%)	*p*	OR (95%CI) *	Cases (%)	Controls (%)	*p*	OR (95%CI) *	Cases (%)	Controls (%)	*p*	OR (95%CI) *
***TNF-α***													
**rs361525**	G	98.04	100	0.05	---	97.89	100	0.03	---	99	99.01	0.995	0.99 (0.10–9.66)
	A	1.96	0	0.05	---	2.11	0	0.03	---	1	0.99	0.995	1.01 (0.10–9.79)
**Codominant**	G/G	96.08	100	0.05	---	95.79	100	0.03	---	98	98.01	0.995	0.99 (0.10–9.77)
	G/A	3.92	0	0.05	---	4.21	0	0.03	---	2	1.99	0.995	1.01 (0.10–9.90)
**rs1800629**	**G**	**89.71**	**96.46**	**0.01**	**0.32 (0.13–0.77)**	**89.47**	**96.23**	**0.01**	**0.33 (0.14–0.378**	96	92.05	0.179	2.07 (0.70–6.12)
	***A***	***10.29***	***3.54***	***0.01***	***3.13 (1.29–7.54)***	***10.53***	***3.77***	***0.01***	***1.00 (1.29–6.98)***	4	7.95	0.179	0.48 (0.16–1.42)
**Codominant**	**G/G**	**79.41**	**92.93**	**0.01**	**0.29 (0.12–0.73)**	**78.95**	**92.45**	**0.01**	**0.31 (0.13–0.73)**	92	84.11	0.162	2.17 (0.72–6.60)
	***G/A***	***20.59***	***7.07***	***0.01***	***3.41 (1.37–8.43)***	***21.05***	***7.55***	***0.01***	***3.27 (1.36–7.82)***	8	15.89	0.162	0.46 (0.15–1.39)
***IL-10***													
**rs1800872**	G	58.33	54.04	0.39	1.19 (0.80–1.77)	57.89	54.72	0.52	1.14 (0.77–1.69)	63	53.97	0.115	1.45 (0.91–2.31)
	T	41.67	45.96	0.39	0.84 (0.57–1.25)	42.11	45.28	0.52	0.88 (0.59–1.30)	37	46.03	0.115	0.69 (0.43–1.09)
**Codominant**	G/G	25.49	25.25	0.97	1.01 (0.54–1.91)	24.21	26.42	0.72	0.89 (0.47–1.68)	32	23.18	0.214	1.56 (0.77–3.15)
	G/T	65.69	57.58	0.24	1.41 (0.79–2.49)	67.37	56.6	0.12	1.58 (0.89–2.81)	62	61.59	0.959	1.02 (0.53–1.97)
	T/T	8.82	17.17	0.08	1.47 (0.19–1.10)	8.42	16.98	0.07	0.45 (0.19–1.09)	6	15.23	0.092	0.35 (0.10–1.24)
**rs1800871**	G	58.33	54.04	0.39	1.19 (0.80–1.77)	57.89	54.72	0.52	1.14 (0.77–1.69)	63	53.97	0.115	1.45 (0.91–2.31)
	A	41.67	45.96	0.39	0.84 (0.57–1.25)	42.11	45.28	0.52	0.88 (0.59–1.30)	37	46.03	0.115	0.69 (0.43–1.09)
**Codominant**	G/G	25.49	25.25	0.97	1.01 (0.54–1.91)	24.21	26.42	0.72	0.89 (0.47–1.68)	32	23.18	0.214	1.56 (0.77–3.15)
	G/A	65.69	57.58	0.24	1.41 (0.79–2.49)	67.37	56.6	0.12	1.58 (0.89–2.81)	62	61.59	0.959	1.02 (0.53–1.97)
	A/A	8.82	17.17	0.08	0.47 (0.19–1.10)	8.42	16.98	0.07	0.44 (0.19–1.09)	6	15.23	0.092	0.36 (0.10–1.24)
**rs1800896**	**T**	**67.65**	**78.28**	**0.02**	**0.58 (0.37–0.91)**	**67.37**	**77.83**	**0.02**	**0.59 (0.38–0.92)**	**58**	**77.81**	**1 × 10^−4^**	**0.39 (0.24–0.64)**
	***C***	***32.35***	***21.72***	***0.02***	***1.72 (1.10–2.69)***	***32.63***	***22.17***	***0.02***	***1.70 (1.09–2.65)***	***42***	***22.19***	***1*** ** × 10^−4^**	***2.54 (1.57–4.11)***
**Codominant**	T/T	44.12	57.58	0.06	0.58 (0.33–1.01)	44.21	56.6	0.08	0.61 (0.35–1.06)	***28***	***58.28***	***2*** ** × 10^−4^**	***0.28 (0.14–0.56)***
	T/C	47.06	41.41	0.42	1.26 (0.72–2.19)	46.32	42.45	0.58	1.16 (0.67–2.04)	***60***	***39.07***	***0.009***	***2.34 (1.22–4.49)***
	***C/C***	***8.82***	***1.01***	***0.01***	***9.48 (1.18–76.33)***	***9.47***	***0.94***	***0.01***	***10.99 (1.37–88.45)***	***12***	***2.65***	***0.008***	***5.01 (1.35–18.56)***
**Recesive**	***C/C***	***8.82***	***1.01***	***0.01***	***9.48 (1.18–76.33)***	***9.47***	***0.94***	***0.01***	***10.99 (1.37–88.45)***	***12***	***2.65***	***0.008***	***5.04 (1.35–18.56)***
	**T/C+T/T**	**91.18**	**98.99**	**0.01**	**0.11 (0.01–0.85)**	**90.53**	**99.06**	**0.01**	**0.09 (0.01–0.73)**	**88**	**97.35**	**0.008**	**0.19 (0.05–0.73)**
***IL-6***													
**rs1800795**	G	93.14	91.92	0.64	1.19 (0.57–2.51)	93.16	91.98	0.65	1.19 (0.56–2.51)	**88**	**94.04**	**0.046**	**0.46 (0.21–1.00)**
	C	6.86	8.08	0.64	0.84 (0.39–1.77)	6.84	8.02	0.65	0.84 (0.39_1.78)	**12**	**5.96**	**0.046**	**2.15 (0.99–4.64)**
**Codominant**	G/G	89.22	83.84	0.26	1.59 (0.70–3.63)	89.47	83.96	0.25	1.62 (0.70–3.74)	82	88.08	0.274	0.61 (0.26–1.48)
	G/C	7.84	16.16	0.07	0.44 (1.79–1.08)	7.37	16.04	0.06	0.42 (0.16–1.05)	12	11.92	0.988	1.00 (0.37–2.69)
	C/C	2.94	0	0.09	---	3.16	0	0.07	---	6	0	0.002	---

* Odds ratio (95% Confidence interval). Bold and italics characters indicate susceptibility. Bold and underlined characters indicate protection. *p*-value with Pearson’s Chi-squared, significant level *p* ≤ 0.05. T2D: Type 2 diabetes mellitus. DR: Diabetic retinopathy. DKD: Diabetic kidney disease.

**Table 4:** Haplotype frequency of *TNF-α*, *IL-10,* and *IL-6* polymorphisms in T2D patients and T2D patients with different microvascular complications.

	T2D with VS. Without Microvascular Complications				T2D with VS. Without DR				T2D with VS. Without DR			
Haplotypes	Cases (%)	Controls (%)	*p*	OR (95%CI) *	Cases (%)	Controls (%)	*p*	OR (95%CI) *	Cases (%)	Controls (%)	*p*	OR (95%CI) *
***TNF-α* rs361525-rs1800629**												
**GG**	**88**	**96.46**	**0**	**0.27 (0.11–0.65)**	**89.68**	**96.23**	**0.002**	**0.28 (0.12–0.65)**	95.8	91.06	0.124	2.23 (0.78–6.42)
***GA***	***9.9***	***3.54***	***0.01***	***2.99 (1.24–7.25)***	***10.32***	***3.77***	***0.012***	***2.87 (1.23–6.70)***	3.2	7.95	0.101	0.38 (0.12–1.26)
***IL-10* rs1800872-rs1800871-rs1800896**												
TAT	41	45.2	0.38	0.84 (0.56–1.24)	41.61	44.87	0.509	0.88 (0.59–1.30)	36.6	45.1	0.137	2.21 (0.44–1.12)
GGT	27	33.08	0.17	0.74(0.48–1.14)	26.33	33.56	0.116	0.71 (0.46–1.09)	**21.4**	**32.72**	**0.032**	**0.56 (0.33–0.96)**
***GGC***	***32***	***20.96***	***0.02***	***1.74 (1.10–2.73)***	***32.06***	***21.58***	***0.018***	***1.71 (1.09–2.68)***	***41.6***	***21.26***	***6 × 10^−5^***	***2.64 (1.63–4.28)***

* Odds ratio (95% Confidence interval). Bold and italics characters indicate susceptibility. Bold and underlined characters indicate protection. *p*-value with Pearson’s Chi-squared, significant level *p* ≤ 0.05. T2D: Type 2 diabetes. DR: Diabetic retinopathy. DKD: Diabetic kidney disease.

**Table 5:** Results from the Wald stepwise backward logistic regression.

	T2D with VS. Without Microvascular Complications		T2D with VS. Without DR		T2D with VS. Without DKD	
Variable	*p*	OR (95%CI) *	*p*	OR (95%CI) *	*p*	OR (95%CI) *
AHT	1.30 × 10^−4^	6.25 (2.45–16.00)	1.23 × 10^−3^	3.95 (1.72–9.09)	5.91 × 10^−4^	6.43 (2.22–18.59)
Smoking	---	---	---	---	0.043	2.66 (1.03–6.85)
Age	2.43 × 10^−3^	1.08 (1.03–1.14)	---	---	0.021	1.06 (1.01–1.11)
T2D Time	8.16 × 10^−5^	0.81 (0.73–0.90)	---	---	1.93 × 10^−3^	0.85 (0.77–0.94)
Weight	1.10 × 10^−7^	1.13 (1.08–1.19)	6.04 × 10^−8^	1.12 (1.08–1.17)	3.67 × 10^−4^	1.13 (1.06–1.22)
BMI	---	---	---	---	1.80 × 10^−3^	0.67 (0.52–0.86)
HbA1C	8.11 × 10^−5^	1.51 (1.23–1.86)	7.86 × 10^−6^	1.52 (1.27–1.83)	3.85 × 10^−5^	1.69 (1.32–2.17)
rs361525AG	---	---	---	---	0.045	0.021 (4.8 × 10^−4^–0.91)
rs1800896CC	1.52 × 10^−2^	32.42 (1.96–537.70)	1.76 × 10^−3^	39.36 (3.94–393.29)	9.47 × 10^−5^	59.46 (7.65–462.41)
rs1800896TC	1.06 × 10^−2^	4.14 (1.39–12.33)	8.55 × 10^−4^	4.08 (1.79–9.33)	4.56 × 10^−4^	7.51 (2.43–23.16)
rs1800795GC	---	---	---	---	1.36 × 10^−2^	7.04 (1.49–33.18)

Independent variables used for analysis: Sex (M/F), Smoking (yes/no) Arterial hypertension (AHT, yes/no), Age (years), T2D time (years), Weight (Kg), BMI (kg/m^2^), Glycosylated hemoglobin (HbA1c, %), Fasting glucose, (mMol/L), LDL (mg/dL), HDL (mg/dL) rs361525 (G/G, A/G), rs1800629 (G/G, A/G), rs1800872 (G/G, G/T, T/T), rs1800871 (G/G, G/A, A/A), rs1800896 (T/T, T/C, C/C). T2D: Type 2 diabetes mellitus. DR: Diabetic retinopathy. DKD: Diabetic kidney disease.

## Data Availability

The raw data supporting the conclusions of this article will be made available by the authors upon request.

## Supplementary Materials

The following supporting information can be downloaded at: https://www.mdpi.com/article/10.3390/pathophysiology32020014/s1, Table S1: Clinical data from this study and from different authors; Table S2: Polymorphism data from this study and from different authors.

## Abbreviations

The following abbreviations are used in this manuscript:

T2D	Type 2 diabetes mellitus
DR	Diabetic retinopathy
DKD	Diabetic kidney disease
SNP	Single nucleotide polymorphism
TNF-α	Tumor necrosis factor-*α*
IL-6	Interleukin-6
IL-10	Interleukin-10
BMI	Body mass index
HbA1c	Glycosylated hemoglobin
AHT	Arterial hypertension
LDL	Low density lipids
HDL	High density lipids
OR	Odds ratio
CI	Confidence interval
